# Activity of BKM120 and BEZ235 against Lymphoma Cells

**DOI:** 10.1155/2015/870918

**Published:** 2015-10-18

**Authors:** Monica Civallero, Maria Cosenza, Samantha Pozzi, Alessia Bari, Paola Ferri, Stefano Sacchi

**Affiliations:** ^1^Program of Innovative Therapies in Oncology and Haematology, Department of Diagnostic and Clinical Medicine and Public Health, University of Modena and Reggio Emilia, Italy; ^2^Department of Diagnostic, Clinical and Public Health Medicine, University of Modena and Reggio Emilia, Italy

## Abstract

Non-Hodgkin lymphomas encompass a heterogeneous group of cancers, with 85–90% arising from B lymphocytes and the remainder deriving from T lymphocytes or NK lymphocytes. These tumors are molecularly and clinically heterogeneous, showing dramatically different responses and outcomes with standard therapies. Deregulated PI3K signaling is linked to oncogenesis and disease progression in hematologic malignancies and in a variety of solid tumors and apparently enhances resistance to antineoplastic therapy, resulting in a poor prognosis. Here, we have evaluated and compared the effects of the pan-PI3K inhibitor BKM120 and the dual PI3K/mTOR inhibitor BEZ235 on mantle, follicular, and T-cell lymphomas. Our results suggest that BKM120 and BEZ235 can effectively inhibit lymphoma cell proliferation by causing cell cycle arrest and can lead to cell death by inducing apoptosis and autophagy mediated by ROS accumulation. Despite great advances in lymphoma therapy after the introduction of monoclonal antibodies, many patients still die from disease progression. Therefore, novel treatment approaches are needed. BKM120 and BEZ235 alone and in combination are very effective against lymphoma cells *in vitro*. If further studies confirm their effectiveness in animal models, they may be promising candidates for development as new drugs.

## 1. Introduction

The phosphatidylinositol 3-kinase [PI3K] signaling pathway plays important roles in many physiological functions, including cell cycle progression, differentiation, survival, motility, apoptosis, autophagy, and protein synthesis [1–4]. PI3K activation leads to phosphorylation of AKT on Thr308, which in turn activates the mammalian target of rapamycin (mTOR), a distal element of the PI3K/AKT/mTOR pathway [5]. mTOR is a serine/threonine kinase that encompasses two distinct complexes—mTORC1 and mTORC2—that differ structurally and in substrate specificity and functionally [6]. mTORC1 induces cell growth in response to nutrients and growth factors by regulating the translational regulators S6K1 and 4E-BP1. mTORC2 mediates cell proliferation and survival by phosphorylating AKT on Ser473, facilitating its full activation [7–10]. The PI3K/Akt/mTOR pathway integrates survival signals from extracellular and intracellular stimuli to promote cell growth and inhibit cell death [11]. This pathway is also involved in regulating autophagy, the ubiquitous and evolutionarily conserved process through which cytosolic components are degraded via lysosomes, helping cells to survive various forms of stress [12]. There remains some debate regarding the precise role that autophagy plays in cancer development, disease progression, and response to anticancer therapies [13]. In contrast to apoptosis, autophagy is initially a survival mechanism triggered when cells are exposed to metabolic stresses. However, recent studies indicate that autophagy and apoptosis are actually interconnected processes [14].

Deregulated PI3K signaling is linked to oncogenesis and disease progression in a variety of solid tumors and hematologic malignancies, apparently enhancing resistance to antineoplastic therapy and resulting in poor prognosis [15]. PI3K discovery and subsequent mapping of the signaling pathway heralded intense efforts to develop inhibitors targeting the pathway components [16–18]. A number of PI3K/AKT/mTOR signaling inhibitors have been developed that selectively interfere with different pathway components and thus exert different biological effects. However, the initial enthusiasm for using PI3K, Akt, or mTOR inhibitors as antineoplastic agents has been tempered by observations that such inhibition typically promotes growth arrest rather than cell death in solid tumors [19].

BKM120 and BEZ235 are synthetic small molecules of the imidazoquinolines class, which show preclinical activity against a range of solid and hematological malignancies. BKM120 is an oral pan-class I PI3-kinase inhibitor belonging to the 2,6-diporpholino pyrimidine derivatives. It selectively inhibits PI3K *α*, *β*, *γ*, and *δ*, as well as mutated PI3K (H1047R, E542K, and E545K). BKM120 reportedly inhibits phosphorylation of the PI3K downstream target Akt and exhibits antiproliferative activity in several cancer cell lines [20–22]. BKM120 shows preclinical activity in distinct malignancies and is currently being tested in a number of clinical trials (ClinicalTrials.gov, U.S. National Institutes of Health, Bethesda, MD, USA: http://clinicaltrials.gov/). BEZ235 is a dual class I PI3K/mTOR inhibitor that competes at its ATP-binding site [23–25]. Increasing evidence demonstrates that BEZ235 effectively and specifically reverses hyperactivation of the PI3K/mTOR pathway, thus exerting potent antiproliferative and antitumor activities in a broad range of cancer cell lines and experimental tumors. It is currently undergoing phase I evaluation in acute leukemia [NCT01756118] [26, 27].

Promising preclinical and clinical data support the rationale for therapeutic use of PI3K/Akt/mTOR inhibitors in lymphoma treatment [28] and, crucially, these inhibitors are well tolerated. However, despite the progress in pharmacological targeting of PI3K signaling, several important issues must be considered. The considerable plasticity within the PI3K signaling pathway makes it unlikely that PI3K inhibitors will provide a universal therapy across the range of diseases in which PI3K has been implicated. Non-Hodgkin lymphomas encompass a heterogeneous group of cancers, with 85–90% arising from B lymphocytes and the remainder deriving from T or NK lymphocytes. These tumors are molecularly and clinically heterogeneous, showing dramatically different responses and outcomes with standard therapies.

The present study aimed at investigating the effects of BKM120 and BEZ235 on mantle, follicular, and T-cell lymphoma cell lines and patients' cells. We evaluated possible new targets and potential strategies for enhancing the anticancer efficacy of these two compounds.

## 2. Material and Methods

### 2.1. Cell Lines

The cutaneous T-cell lymphoma cell line HUT78 and the mantle cell lymphoma cell line GRANTA519 were purchased from Deutsche Sammlung von Mikroorganismen und Zellkulturen GmbH and were characterized as specified (https://www.dsmz.de/research/human-and-animal-cell-lines.html). The B-cell lymphoma cell line WSU-NHL was kindly provided by Dr. M. Introna (Ospedale Riuniti, Bergamo, Italia). All cell lines were cultured in RPMI-1640 supplemented with 10% fetal bovine serum (FBS), 2 mM L-glutamine, and 100 U/mL penicillin and streptomycin (all purchased from Euroclone). Cells in logarithmic growth phase were used for experiments.

### 2.2. Patient Samples

Peripheral blood mononuclear cells (PBMCs) were obtained from three patients with mantle cell lymphoma, four patients with follicular lymphoma, two patients with T-cell lymphoma, and four authors of the paper who volunteered. All donors gave their informed consent, and this study protocol was approved by the local ethics committee of University of Modena and Reggio Emilia.

### 2.3. Drugs

BKM120 was a gift from Novartis Pharma. BEZ235 and everolimus were purchased from Selleck Chemicals. Chloroquine, paclitaxel, and nocodazole were purchased from Sigma Aldrich (St. Louis, MO, USA). BKM120, BEZ235, everolimus, and paclitaxel were each dissolved in dimethylsulfoxide (DMSO; Euroclone) to create 10^−2^ M stock solutions that were stored at −80°C. The stock solutions were further diluted with cell culture medium to appropriate concentrations before use. The maximum final concentration of DMSO (<0.1%) did not affect cell proliferation and did not induce cytotoxicity on the tested cell lines and primary cells (data not shown).

### 2.4. Assessment of Viability and Cell Proliferation

Cell lines were treated with increasing concentrations of drugs for 24 and 48 h. Viability was assessed by exclusion assay with 0.2% Trypan Blue (Euroclone), and cell proliferation was monitored by MTT assay (CellTiter nonradioactive cell proliferation assay; Promega) as previously described [29]. The drug concentration required for 50% inhibition of cell proliferation (IC_50_) was evaluated with Calcusyn software (Biosoft, Cambridge, UK) applying the median-effect method [30].

### 2.5. Annexin IV Binding Assay for Apoptosis

Apoptosis was quantified using an annexin/propidium iodide (PI) binding assay following the manufacturer's instructions (Miltenyi Biotec). Events were acquired using a FACSCalibur cytometer (Becton Dickinson) and then analyzed using FlowJo Software (Tree Star, Ashland, OR, USA).

### 2.6. Caspase-3 Activity Assay

Caspase-3 activity was measured using a colorimetric substrate for caspase-3 (CPP32) (Enzo Life Sciences) with a sequence based on poly(ADP ribose) polymerase (PARP) cleavage site Asp216 for caspase-3. The full-length and the cleaved procaspase-3 were incubated with the substrate for the indicated times, and the cleavage rate was colorimetrically monitored at 405 nm.

### 2.7. Autophagy Detection

To measure autophagy in cell lysates, we used the quantitative immunometric detection method provided by the p62 (sequestosome 1) ELISA kit, following the manufacturer's instructions (Enzo Life Science, Farmingdale, NY, USA).

### 2.8. Cell Cycle Analysis

Cell cycle analysis was performed using propidium iodide (PI) staining (Miltenyi Biotec) and a FACSCalibur cytometer. The percentages of cells in the subG1/G0 (dead cells), G1/G0, S, and G2/M phases were calculated using Modfit LT software (Verity Software House, Topsham, ME, USA).

### 2.9. Analysis of Tubulin Expression

Cells were seeded at 100,000 cells/well in 24-well plates in RPMI-1640 with 10% FBS, 100 *μ*g/mL penicillin, 100 U/mL streptomycin, and drugs at the indicated concentrations. After incubation for different time durations, the cells were resuspended in 0.5 mL microtubule-stabilizing buffer (80 mM Pipes, 1 mM MgCl_2_, 5 mM EDTA, and 0.5% Triton X-100) containing 0.5% glutaraldehyde at pH 6.8. After a 10 min incubation at room temperature, glutaraldehyde was quenched by addition of 0.5 mL of 0.25 M glycine solution in PBS. Cells were then spun at 1000 g for 7 min, resuspended in 20 *μ*L of 50 *μ*g/mL RNase A in antibody diluting solution (Ab-Dil; PBS, 0.2% Triton X-100, 2% bovine serum albumin, and 0.1% NaN_3_; pH 7.4), and incubated overnight at 4°C. The next day, the cells were incubated with *α*-tubulin (1 : 50) in Ab-Dil for 3 h at room temperature and then with anti-rabbit IgG1-FITC. Finally, the cells were further diluted in 500 *μ*L FACS buffer (PBS, 2% BSA, and 0.1% sodium azide; pH 7.2) followed immediately by flow cytometry analysis. For each sample, the mean fluorescence intensity was recorded using a FACSCalibur cytometer and analyzed using FlowJo Software. Tubulin levels were determined based on the geometric mean of the antibody/FITC fluorescence and were normalized to a value of 100 for the vehicle control. All of the abovementioned reagents were purchased from Sigma Aldrich.

### 2.10. Western Blot Analysis

After drug treatments, the cell lines were harvested and lysed using the Mammalian Cell Extraction Kit (BioVision, Milpitas, CA, USA) following the manufacturer's instructions. Proteins (100 *μ*g/lane) were electrophoresed on 4–20% SDS-polyacrylamide gradient gels and transferred to nitrocellulose membranes (Bio-Rad Laboratories, Hercules, CA, USA). Membranes were immunoblotted with the following primary antibodies: total AKT, p-AKT (ser 473), total p70S6, p-p70S6 (Thr421/Ser424), total m-TOR, p-m-TOR (ser 2448), total EBP1, p-EBP1 (Thr37/46), cyclin D, cyclin E, cyclin A, p-21 (Waf1/Cip1), p-27 (Thr187), Aurora A, p-BAD (Ser112), PUMA, BIM, MCL, BCL-XL, BCL-2, caspase-3 (Asp175), caspase-9 (Asp353), caspase-8 (Asp391), PARP, and LC3. All of the abovementioned antibodies were diluted 1 : 1000 and purchased from Cell Signaling Technology (Danvers, MA, USA). Then, the membranes were incubated with species-specific horseradish peroxidase- (HRP-) conjugated secondary antibody (1 : 500) from Cell Signaling Technology. Blots were developed using SuperSignal West Pico Chemiluminescent Substrate (Thermo Scientific, Rockford, IL, USA). Images were acquired by Chemidoc XRS+ and analyzed using Image Lab Software v.3.0 (Bio-Rad Laboratories).

### 2.11. Measurement of Reactive Oxygen Species (ROS) Production

To determine ROS production, cells were incubated with 20 *μ*M/L 2′,7′-dichlorodihydrofluorescein diacetate (DCFH-DA) (Sigma Aldrich) in complete medium for 30 minutes at 37°C. The extent of ROS generation was measured by quantifying the fluorescence with a FACSCalibur cytometer (Becton Dickinson), and these data were analyzed using FlowJo Software (Tree Star).

### 2.12. Combination Study

Cell lines were seeded at 5 × 10^4^ cells per well in 96-well plates. Each experiment was performed in triplicate. Cells were cultured in the absence or presence of drugs, individually or in combination, at concentrations below the IC_50_. The inhibitory effects of drug combination treatments were determined using the MTT assay as described above. Drug interactions were assessed using multiple effect analysis, based on the method described by Chou-Talalay in which a combination index (CI) of <1 indicates synergism, 1 indicates additive effects, and >1 indicates antagonism [31]. The data were processed by isobologram analysis using Stata 8.2.

### 2.13. Statistical Analysis

All experiments were independently repeated three times, with multiple replicates within each run. Data are expressed as mean ± standard error. Statistical differences between control and drug-treated cells were analyzed by one-way ANOVA and by two-tailed Student's test. Data were analyzed using the Stata 8.2/SE package (StataCorp LP, College Station, TX, USA). Correlation coefficients (*r*) and the statistical significance of differences between cohorts were determined using Prism's correlation analysis based on two-tailed *p* values (GraphPad Software, USA), with *p* values of <0.01 considered statistically significant.

## 3. Results

### 3.1. Growth Inhibition

**Figure 1:**
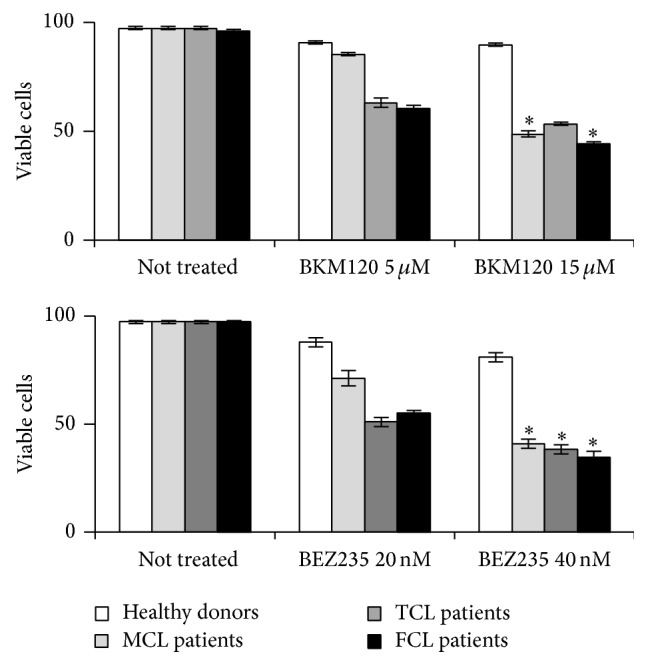
Antiproliferative effects of BKM120 and BEZ235 on PBMCs from three patients with mantle cell lymphoma (MCL), four patients with follicular cell lymphoma (FCL), two patients with T-cell lymphoma (TCL), and four healthy donors. Cells were treated with BKM120 (5 *μ*M and 15 *μ*M) and BEZ235 (20 nM and 40 nM) for 24 hours. Viability was assessed using 0.2% Trypan Blue exclusion. Values represent mean ± SD and were obtained from three independent experiments performed in triplicate. ^*∗*^ *p* < 0.01 compared with control.

To determine the IC_50_ values and the effects of the drugs on cell viability, lymphoma cell lines were cultured with increasing concentrations of BKM120 (0.5–15 *μ*M) and BEZ235 (0.5–50 nM) for 24 and 48 hours.  Table 1 shows the IC_50_ values obtained for BKM120 and BEZ235 in HUT78, GRANTA519, and WSU-NHL cell lines. Results were obtained from three independent experiments performed in triplicate. We also evaluated the drugs' effects on PBMCs from three patients with mantle cell lymphoma, four patients with follicular lymphoma, two patients with T-cell lymphoma, and four healthy donors (Figure 1). We observed that BKM120 and BEZ235 each significantly and dose-dependently decreased the percentage of viable PBMCs from patients with lymphoma but had minimal or no cytotoxic effect on PBMCs from healthy donors. We treated the cells for only 24 hours because 48 hours of treatment was too toxic (data not shown).

### 3.2. Cell Cycle

**Figure 2:**
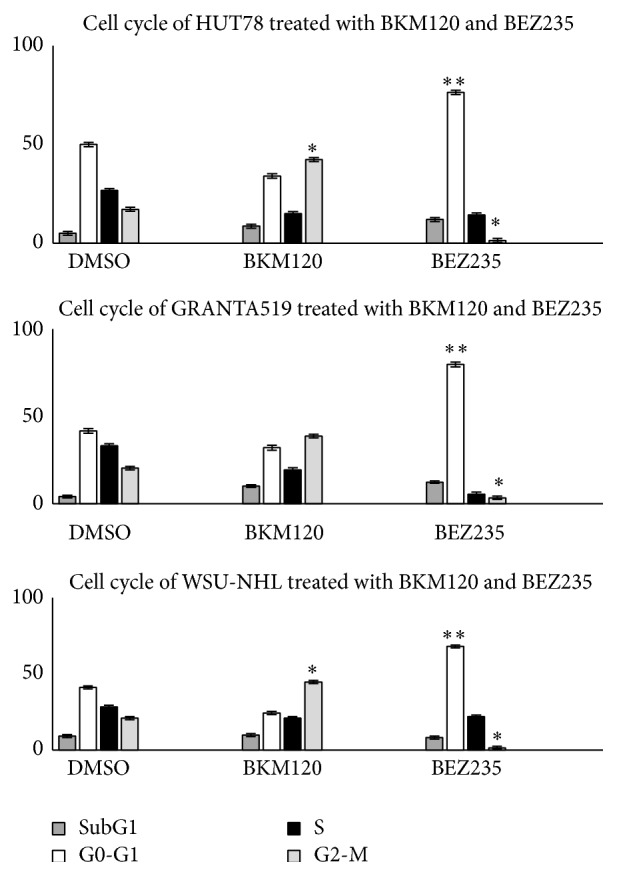
Cell cycle profiles of HUT78, GRANTA519, and WSU-NHL cells after 24 hours of treatment with the IC_50_ of BKM120 and BEZ235. Data are expressed as mean ± SD, obtained from three independent experiments performed in triplicate. ^*∗*^ *p* < 0.01 compared with control.

**Figure 3:**
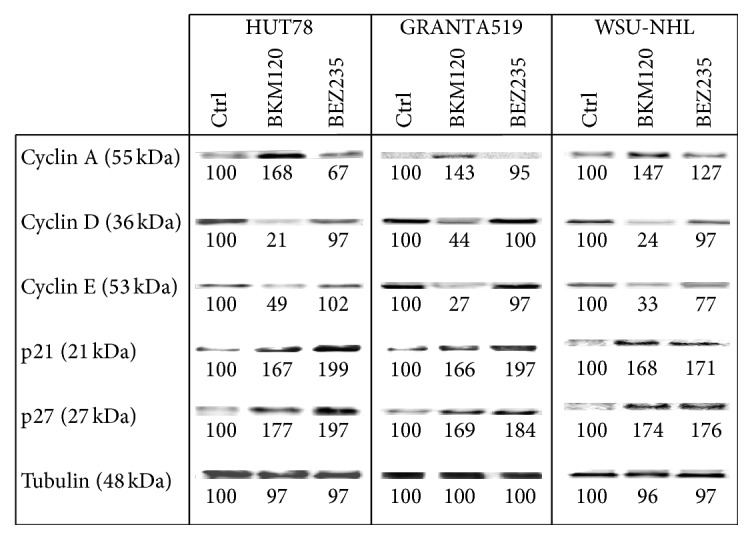
Western blots of cellular extracts from HUT78, GRANTA519, and WSU-NHL cell lines treated with the IC_50_ of BKM120 and BEZ235 for 24 h. Cellular extracts were probed with antibodies against cyclin A, cyclin D, cyclin E, p21, and p27. Densitometric semiquantification of bands normalized to the untreated control is shown below the immunoblot bands.

We employed flow cytometric analysis to determine whether 24 hours of treatment with the IC_50_ of BKM120 and BEZ235 led to cell cycle progression defects (Figure 2). To investigate the effects of drugs on cell cycle-related proteins, we used western blot analysis to evaluate the expressions of cyclin A, cyclin D, cyclin E, p21, and p27 (Figure 3). BKM120 induced a dose-dependent increase of G2 phase with downregulation of cyclin D and cyclin E and upregulation of cyclin A, p21, and p27. On the other hand, BEZ235 induced an increase of G0/G1 phase, with downregulation of cyclin A and upregulation of cyclin D, cyclin E, p21, and p27.

**Figure 4:**
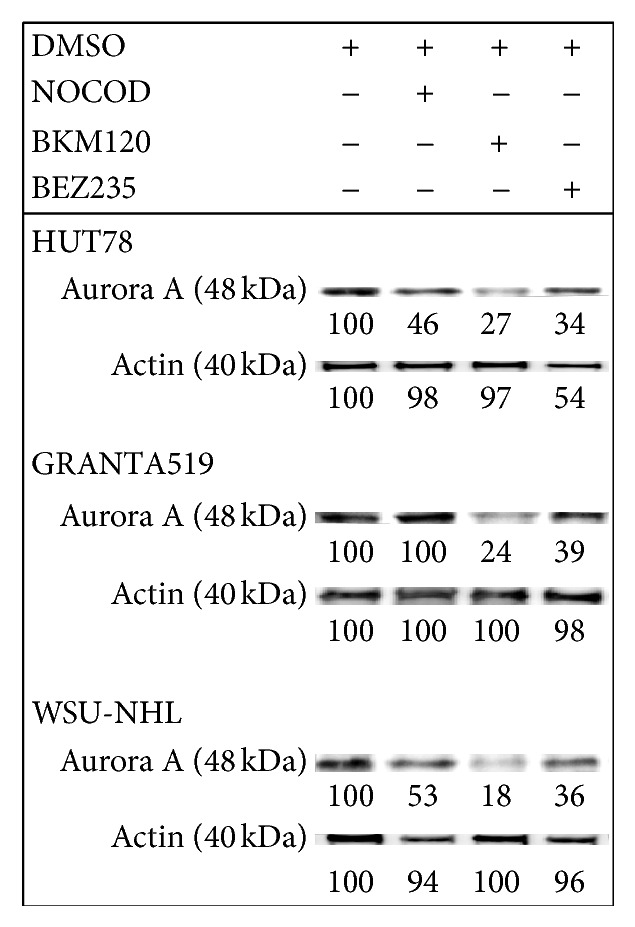
Western blot of cellular extracts from HUT78, GRANTA 519, and WSU-NHL. The cells were treated (1) for 24 with 1 *μ*M nocodazole (NOCOD) and (2) for 24 with the IC_50_ of BKM120 and BEZ235. Cellular extracts were probed with antibodies against Aurora A. Densitometric semiquantification of bands normalized to the untreated control is shown below the immunoblot bands.

Immunoblotting revealed that all tested cell lines expressed Aurora A kinase protein, which regulates cell-cycle checkpoints and cell cycle regulatory molecules (Figure 4) [32]. We next determined whether BKM120 and BEZ235 inhibited Aurora A kinase expression in lymphoma cell lines. To determine the compounds' inhibitory effects on the mitotic cell population, we first synchronized cell division in lymphoma cell lines by treatment with nocodazole (1 *μ*M) for 12 hours. The cells were then washed and treated with BKM120 and BEZ235 for 24 h. Aurora A kinase phosphorylation was measured by western blotting. Decreased Aurora A kinase expression was observed in all cell lines treated with BKM120, compared with in the DMSO and nocodazole-treated cells.

### 3.3. Drug-Cell Correlations for Tubulin Assembly, Cycle Arrest, and Cell Viability

Our findings indicated that BKM120 induced cell cycle arrest in G2, while BEZ235 induced arrest in G1. By further analyzing intracellular tubulin polymerization kinetics, cell cycle G2 population arrest, and cell viability in dose response mode, we obtained a comprehensive understanding of the drugs' intracellular mechanisms of action at a molecular level. We utilized a cytometric-based technique that allows direct quantitative evaluation of tubulin/microtubule dynamics without interference from microtubule-associated proteins or other complicating factors [33], thus enabling facile comparison of compounds that affect tubulin polymerization.

**Figure 5:**
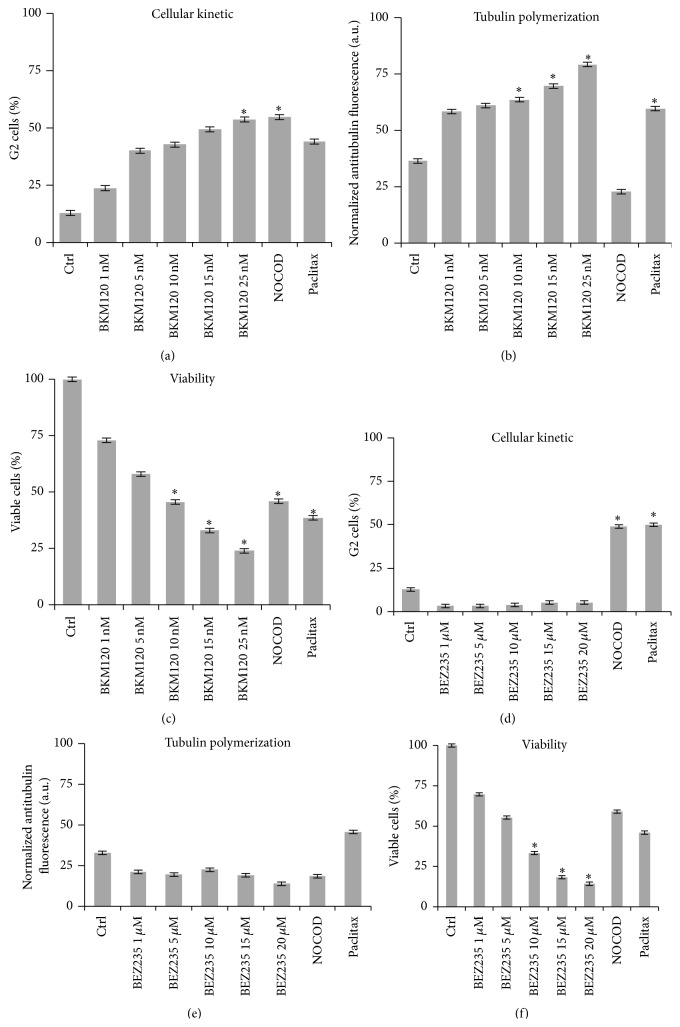
HUT78 cells were treated for 24 hours with increasing concentrations of BKM120 and BEZ235 as well as with the microtubule destabilizer nocodazole (1 *μ*M) and the microtubule stabilizer paclitaxel (100 nM). Representative data from analyses of cell cycle arrest in G2 (a), tubulin polymerization (b), and cell viability (c) after BKM120 treatment. Representative data from analyses of cell cycle arrest in G2 (d), tubulin polymerization (e), and cell viability (f) after BEZ235 treatment. Data are expressed as mean ± SD and were obtained from three independent experiments performed in triplicate (Nocod = nocodazole; Paclitax = paclitaxel) ^*∗*^ *p* < 0.01 compared with control.

HUT78, GRANTA519, and WSU-NHL were treated for 24 hours with increasing BKM120 concentrations along with the microtubule destabilizer nocodazole (1 *μ*M) and the microtubule stabilizer paclitaxel (100 nM). This was followed by whole cell-based qualitative measurement of tubulin polymerization using alpha-tubulin staining.  Figure 5(a) shows that BKM120 induced concentration-dependent progressive induction of G2 arrest in HUT78 cells. Nocodazole and paclitaxel increased the G2 cell population arrest. In Figure 5(b), the fluorescence reflecting cell tubulin assembly revealed similar kinetics, as G2 cell population arrest was observed after treatment with increasing concentrations of BKM120 and after treatment with the tubulin stabilizer paclitaxel. Nocodazole had the opposite effect, as treatment with this tubulin destabilizer clearly decreased tubulin polymerization. Our results showed a positive correlation (*r* > 0.7) between G2 cell population and tubulin assembly following treatment with increasing doses of BKM120.  Figure 5(c) shows a strong negative correlation between tubulin assembly and cell viability (*r* = −0.951). Comparable results were obtained with GRANTA519 and WSU-NHL (data not shown).

HUT78 cells treated with increasing doses of BEZ235 showed a decrease in the G2 population, accompanied by a parallel but very modest action on tubulin polymerization, represented by a negative correlation index (*r* = −0.89) (Figures 5(d) and 5(e)). Cell viability decreased in a dose-dependent manner, which was not correlated with tubulin assembly (*r* = −0.97) (Figure 5(f)). Comparable results were obtained with GRANTA519 and WSU-NHL (data not shown).

### 3.4. Signaling Pathways

**Figure 6:**
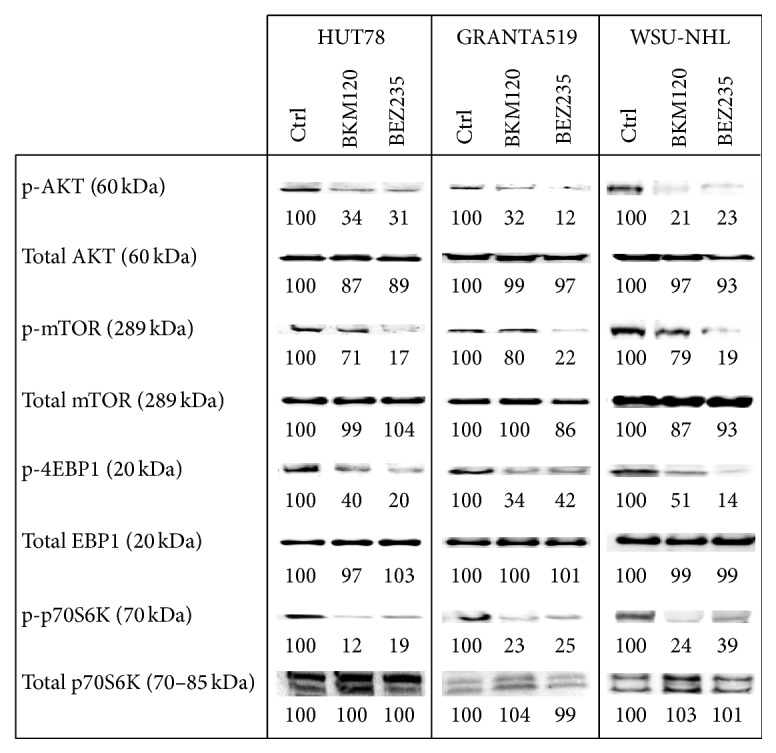
Western blots of cellular extracts from HUT78, GRANTA519, and WSU-NHL that were treated with the IC_50_ of BKM120 and BEZ235 for 24 h. Cellular extracts were probed with antibodies against p-AKT (Ser473), total AKT (Ser473), p-mTOR (Ser2448), total mTOR, p-4EBP1 (Thr37/46), total 4EBP1, p-p70S6kinase (Thr421/Ser424), and total p70S6kinase. Densitometric quantification of bands normalized to the untreated control is shown below the immunoblot bands.

To evaluate the effects of BKM120 and BEZ235 on PI3K/AKT/mTOR signaling, we analyzed the phosphorylation status of Akt and some downstream targets (including mTOR, 4EBP1, and p70S6kinase) in lymphoma cell lines treated for 24 h with the IC_50_ of these drugs. BEZ235 evidently reduced the expressions of PI3K/AKT/mTOR pathway components in all cell lines (Figure 6). In the same cellular lysates, we also evaluated the total expressions of the corresponding proteins (data not shown).

### 3.5. Apoptosis

**Figure 7:**
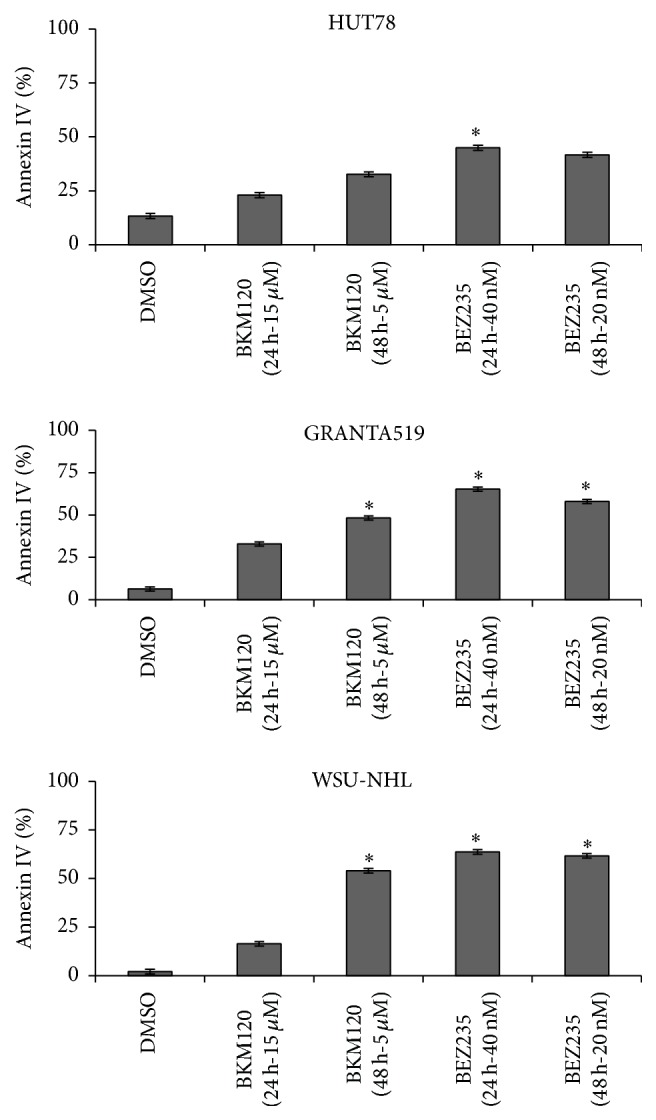
Cell lines were treated for 24 and 48 h with BKM120 and BEZ235 at IC_50_. Apoptotic cells were quantified using annexin IV/PI staining. Each compound dose- and time-dependently induced increased apoptosis, evidenced by annexin IV positive cells. Results represent the mean ± SD obtained from three independent experiments. ^*∗*^ *p* < 0.01 compared with control.

We next examined the functional effects of BKM120 and BEZ235 on apoptosis in lymphoma cell lines. Cells were treated with the IC_50_ of BKM120 and BEZ235 for 24 h and 48 h. Apoptotic cells were quantified using annexin IV/PI staining. Both drugs induced significantly increased apoptosis even at only 24 hours (*p* < 0.01) (Figure 7).

**Figure 8:**
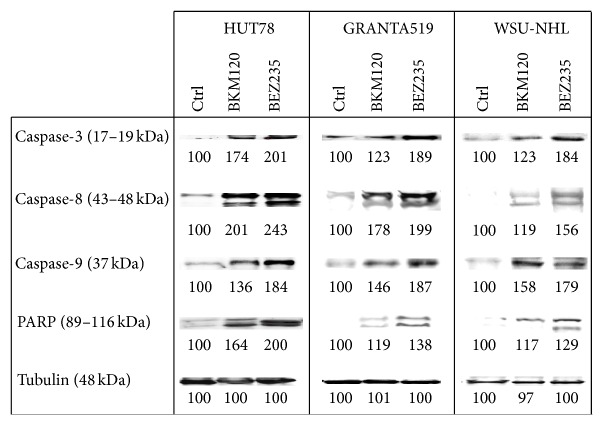
Western blots of cellular extracts from HUT78, GRANTA519, and WSU-NHL cell lines treated for 24 h with the IC_50 _of BKM120 and BEZ235. Cellular extracts were probed with antibodies against the cleaved forms of caspase-3 (Asp175), caspase-8 (Asp391), caspase-9 (Asp535), and PARP (total full length and cleaved fragment).

After flow cytometric analysis, we further attempted to define the mechanisms by which BKM120 and BEZ235 induced apoptosis. We demonstrated that 24 hours of treatment with either compound at IC_50_ induced apoptosis via both intrinsic and extrinsic apoptotic pathways, as demonstrated by caspase-3, caspase-8, caspase-9, and PARP cleavage (Figure 8). To confirm that apoptosis was mediated by activation of these caspases, we cultured the three cell lines in the presence of the broad caspase inhibitor ZVAD-fmk. Notably, NVP-BEZ235 induces cleavage of PARP + zvad-fmk (data not shown).

**Figure 9:**
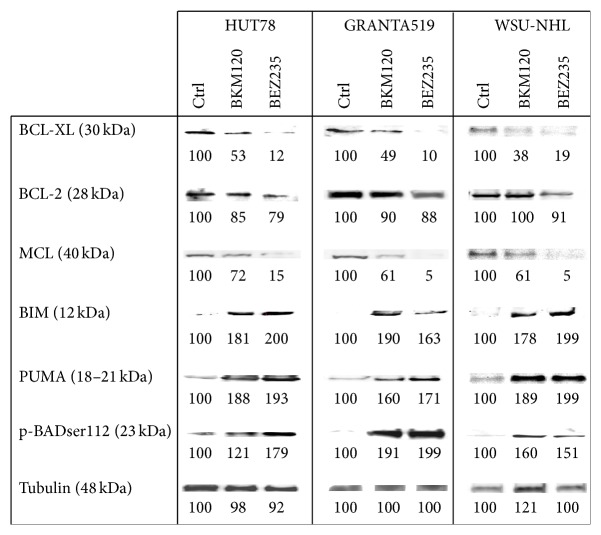
Western blots of cellular extracts from HUT78, GRANTA519, and WSU-NHL cell lines treated for 24 h with the IC_50_ of BKM120 and BEZ235. Cellular extracts were probed with antibodies against BCL-XL, BCL-2, MCL, BIM, PUMA, and p-BADser112.

To further analyze the mechanism of apoptosis induced by BKM120 and BEZ235 inhibition, we measured the expressions of pro- and antiapoptotic members of the BCL-2 family, both before and after treatment with these two compounds. In all tested lymphoma lines, drug treatment led to increases of the proapoptotic proteins (Bim, Bad, and PUMA) and downregulation of the antiapoptotic proteins (BCL-XL and MCL-1) (Figure 9). BCL-2 expression resulted minimally affected by BKM120 and BEZ235 treatment.

### 3.6. Autophagy in Lymphoma Cells

**Figure 10:**
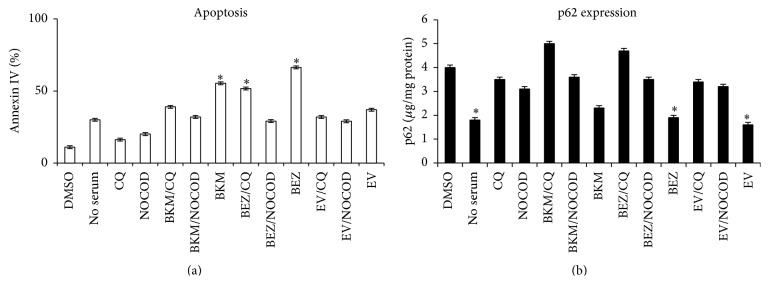
WSU-NHL cells were treated for 24 h with BKM120, BEZ235, and everolimus alone and in combination with CQ. These cells were then analyzed for apoptosis by annexin IV staining (a) and p62 expression (b). We use a serum-free medium to create stress. Results represent the mean ± SD obtained from three independent experiments. ^*∗*^ *p* < 0.01 compared with control.

Aside from its role in regulating cell growth and proliferation, the PI3K/AKT/mTOR pathway also participates in autophagy signaling. Therefore, we investigated whether BKM120, BEZ235, and everolimus induced autophagy. Everolimus is an mTOR inhibitor and therefore an autophagy activator [34, 35]. To do this, we treated all cell lines for 24 h with the IC_50_ of BKM120, BEZ235, and everolimus, both alone and in combination with the autophagy inhibitor chloroquine (CQ) and the inhibitor of autophagosome-lysosome fusion nocodazole [36]. We used the p62 ELISA kit to evaluate the amounts of p62 protein (which accumulates when autophagy is inhibited) in cell lysates, thus allowing quantitative comparison of how HUT78 cells were impacted by the various treatments (Figure 10(a)). BKM120, BEZ235, and everolimus induced autophagy comparable to that which occurs under nutrient deprivation conditions. In the same samples used to study autophagy, we also evaluated cellular apoptosis (Figure 10(b)). BKM120 and BEZ235 alone increased annexin-positive cells, suggesting that the two drugs induced both apoptosis and autophagy. CQ and nocodazole alone inhibited autophagy and did not induce apoptosis, but their cooperative interaction with BKM120 and BEZ235 triggered apoptosis, as confirmed by annexin IV/PI staining. Everolimus induced autophagy but did not induce substantial apoptosis. Comparable results were obtained with GRANTA519 and WSU-NHL (data not shown).

**Figure 11:**
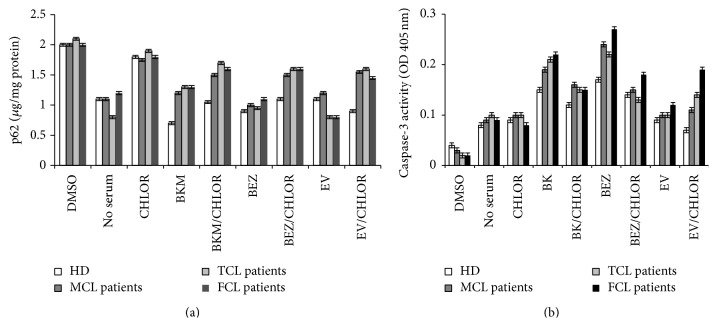
We quantitatively determined p62 expression (a) and apoptosis (based on caspase-3 expression) (b) in PBMCs from patients and donors. We evaluated the effects of BKM120 and BEZ235 on PBMCs from three patients with mantle cell lymphoma (MCL), four patients with follicular cell lymphoma (FCL), two patients with T-cell lymphoma (TCL), and four healthy donors (HD). Results represent the mean ± SD.

As a marker of autophagy, we also monitored lipidation of LC3II by performing western blotting in all cell lines following treatment with BKM120, BEZ235, and everolimus alone and in combination with CQ (data not shown). We also quantitatively determined p62 expression and apoptosis (based on caspase-3 expression) in patients' cells. In PBMCs from patients and donors, we evaluated the effects of BKM120, BEZ235, and everolimus both alone and in combination with CQ (Figures 11(a) and 11(b)). As observed in the cell lines, BKM120 and BEZ235 induced autophagy and apoptosis in the patient and donor cells. Everolimus caused autophagy but did not induce substantial apoptosis. CQ inhibited autophagy and did not induce substantial apoptosis.

### 3.7. ROS Expression

**Figure 12:**
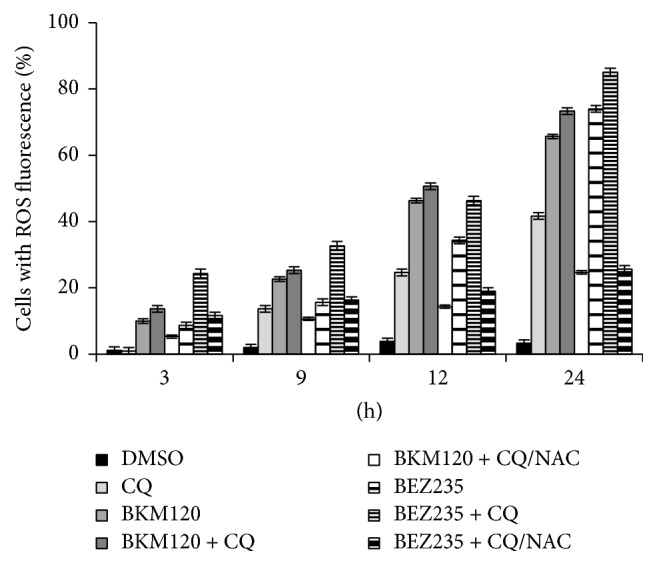
ROS generation in cells was monitored during treatment with BKM120 and BEZ235 alone and in combination with CQ after 3, 9, 12, and 24 hours. The ROS level was determined by flow cytometry, and quantitative analysis of ROS generation is shown in histograms. Coadministration of the antioxidant N-acetyl cysteine (NAC) blocked the increased ROS generation. Results represent the mean ± SD obtained from three independent experiments.

Autophagy and apoptosis have been shown to be regulated by numerous aspects of glucose metabolism and can also be activated by mitochondrial dysfunction and oxidative stress [37]. Here we monitored the generation of reactive oxygen species in cells during treatment with BKM120 and BEZ235 alone and in combination with CQ. Our results showed that BKM120 and BEZ235 alone each induced a higher percentage of cells with ROS generation. This induction was enhanced when these drugs were combined with CQ. Coadministration of the antioxidant N-acetyl cysteine (NAC) blocked the increased ROS generation (Figure 12).

### 3.8. Combination Study

To determine if this combination was additive or synergistic, the interaction between BKM120 and BEZ235 in combination with CQ was investigated applying isobologram analysis based on the Chou-Talalay method. This analysis was performed using STACorp 8.2 software. We found that the interaction between BKM120 and CQ showed moderate synergism (0.5 < CI > 1), while the combination of BEZ235 and CQ was highly synergistic (CI index < 0.5).

## 4. Discussion

PI3K inhibitors that are currently under development are grouped based on their specificity—including pure PI3K inhibitors, compounds that block both PI3K and mTOR (dual inhibitors), pure catalytic mTOR inhibitors, and inhibitors that block Akt. However, it remains unclear which of these inhibitor types is more clinically effective. Numerous PI3K-targeted compounds, many of which are dual PI3K and mTOR inhibitors, are currently being tested in clinical trials [38, 39].

Here, we evaluated and compared the effects of the pan-PI3K inhibitor BKM120 and the dual PI3K/mTOR inhibitor BEZ235 on mantle, follicular, and T-cell lymphomas. Both drugs effectively induced cytotoxicity in lymphoma cell lines and patients' cells. BKM120 and BEZ235 each blocked the PI3K/Akt/mTOR pathway and the downstream targets p90RSK and pEBP1, resulting in growth arrest. Our findings highlight an interesting difference in the actions of the two drugs with regard to blocking the cell cycle. Treatment with BKM120 led to a moderate amount of cell cycle arrest in the G2-M phase in the tested cell lines. In contrast, BEZ235 treatment resulted in substantial dose-dependent accumulation in the G0-G1 phase of the cell cycle. These results were in agreement with the measured expression levels of cyclins A, D, and E and the cycle regulators p21 and p27. The two inhibitors' different roles in regulating the cell cycle could be related to their actions on Aurora A kinase expression. Aurora A kinase belongs to a family of mitotic serine/threonine kinases that are implicated in important processes during mitosis and meiosis [40, 41]. Treatment of our lymphoma cells with BEZ235 led to a decrease in Aurora A expression, while BKM120 did not alter Aurora A kinase expression, supporting a possible key role of this kinase in cell cycle control.

Since BKM120 and BEZ235 showed different roles in regulating cell cycle blockade, we hypothesized that the two inhibitors could interfere with microtubule dynamics of the normal polymerization/depolymerization cycle, resulting in cell cycle arrest. Microtubule stabilizers (such as paclitaxel and derivate) and destabilizers (such as nocodazole) activate the spindle assembly checkpoint, leading to cell arrest in mitosis and subsequent cell killing [42]. Our analysis of tubulin expression in lymphoma cell lines revealed that BKM120 acted as a microtubule stabilizer, with an effect similar to paclitaxel. In contrast, BEZ235 exhibited an action similar to that of the microtubule destabilizer nocodazole. Further structural studies are needed to elucidate how BKM120 and BEZ235 bind to tubulin.

The PI3k/AKT/mTOR pathway plays important roles in apoptosis and autophagy. When assessing whether BEZ235- and BKM120-induced cell death was mediated through apoptosis, we noticed the cleavage of caspases 3, 8, and 9, suggesting activation of both the intrinsic and extrinsic pathways. Indeed, we observed that apoptotic events were sustained by upregulation of proapoptotic factors (e.g., BIM, BAD, and PUMA) and we detected downregulation of the antiapoptotic factors MCL-1 and BCL-XL. Our results highlight a very interesting relationship between apoptosis and autophagy. BKM120 and BEZ235 treatments induced autophagy while triggering apoptosis. Autophagic machinery-targeted agents are emerging as promising tools for lymphoma treatment, and autophagy induction has emerged as a new potential therapeutic avenue to circumvent cancer cells' resistance to cell death [43–45]. However, not all inhibitors of PI3K-Akt-mTOR signaling synergize with autophagy inhibitors. The allosteric mTORC1 inhibitor rapamycin does not induce apoptosis in conjunction with autophagy blockade, due to feedback-activation of Akt [46].

To better understand the effectiveness of drugs on autophagocytosis, we treated all lymphoma cell lines with BKM120, BEZ235, and the rapamycin derivate everolimus, both alone and in combination with the autophagy inhibitor chloroquine. Chloroquine is widely used as an effective and safe antimalarial and antirheumatoid agent. Accumulating lines of evidence suggest that CQ can effectively sensitize cells to the effects of conventional cancer therapies (e.g., ionizing radiation and chemotherapeutic agents) in a cancer-specific manner, enhancing the treatment efficacy [47]. Our results showed that both BKM120 and BEZ235 in combination with CQ cooperated to trigger apoptosis in lymphoma cells. The potency of these cotreatments was underscored by the calculated combination index, demonstrating that both compounds cooperated synergistically.

Accumulating evidence suggests that cancer cells are under increased oxidative stress and therefore more vulnerable to damage by ROS insults induced by exogenous agents [48]. Reactive oxygen species (ROS) are generated as by-products of normal physiological processes. The rate of mitochondrial ROS production can vary depending on cell physiopathological conditions. However, in any case, only some ROS produced within mitochondria are released in the cytosol [37]. A moderate ROS increase can promote cell proliferation and differentiation, whereas excessive ROS can interfere with cellular signaling pathways by causing oxidative damage to lipids, proteins, and DNA [49]. Our results showed that both BKM120 and BEZ235 alone induced a higher percentage of lymphoma cells with ROS generation, and this increase was enhanced when these drugs were combined with CQ. We also studied phagocytosis in patients' cells and the results were comparable to those obtained in the cell lines. Finally, we did not observe differences between the different lymphoma types. In conclusion, our results suggest that BKM120 and BEZ235 can effectively inhibit lymphoma cell proliferation by causing cell cycle arrest and can lead to cell death by inducing apoptosis and autophagy mediated by ROS accumulation.

## 5. Conclusions

Despite great advances in lymphoma therapy following the introduction of monoclonal antibodies, many patients still die from disease progression. Therefore, novel treatment approaches are needed. BKM120 and BEZ235 alone and in combination show high effectiveness against lymphoma cells* in vitro*. If future studies confirm their effectiveness in animal models, these drugs may be promising candidates for development as new drugs.

## Figures and Tables

**(a):** 

BKM120 (*μ*M)	IC_50_ 24 h	IC_50_ 48 h
HUT78	15.1	4.8
	(IC_95%_: 14.8–15.4)	(IC_95%_: 4.6–5.1)

GRANTA519	12.4	3.9
	(IC_95%_: 12.1–12.7)	(IC_95%_: 3.4–4.2)

WSU-NHL	14.8	4.1
	(IC_95%_: 14.4–15.5)	(IC_95%_: 3.8–4.6)

**(b):** 

BEZ235 (nM)	IC_50_ 24 h	IC_50_ 48 h
HUT78	41.6	21.1
	(IC_95%_: 41.3–41.9)	(IC_95%_: 20.9–22.4)

GRANTA519	45.1	25.3
	(IC_95%_: 44.8–46.2)	(IC_95%_: 25.2–25.5)

WSU-NHL	39.2	18.5
	(IC_95%_: 39.01–39.5)	(IC_95%_: 18.2–18.8)
